# The Impact of Immigration Status on the Experience of Obstetric Care in Quebec

**DOI:** 10.1007/s10903-025-01809-4

**Published:** 2025-11-14

**Authors:** Rania Ammar, Sylvie Lévesque, Isabelle Boucoiran, Anna Medvetskaya, Arianne Jean-Thorn, Natacha Godbout

**Affiliations:** 1 School of Public Health, University of Montreal, Montreal, Canada; 2 Research Center of CHU Sainte-Justine, Montreal, Canada; 3 Department of Sexology, UQAM, Montreal, Canada; 4 Department of Obstetrics and Gynecology, University of Montreal, Montreal, Canada; 5 Department of Psychology, UQAM, Montreal, Canada

**Keywords:** Obstetric care, Childbirth, Quebec, Immigrants, Mistreatment, Satisfaction, Temporary resident

## Abstract

To compare the experience of obstetric care in Quebec between immigrants and Canada-born persons.A cross-sectional survey was conducted among individuals who received obstetric care in Quebec between 2016 and 2023. Participants, recruited mainly via social media, completed an online questionnaire between July and December 2023. Obstetric care experiences, including autonomy measured by the MADM scale (Mothers Autonomy in Decision Making), mistreatment assessed by the MIST index (Pregnant Persons Experience of Mistreatment by Providers Index), discrimination, access to care, and satisfaction towards interpersonal skills of healthcare providers, were compared between immigrant and Canada-born participants. Among 686 participants, 11.2% were immigrants, among which 69.0% had permanent status and 14.3% temporary status. There was no significant difference in MADM scale between immigrant and Canada-born participants (*p* = 0.903), but immigrants with temporary status were 9.25 times more likely to have low autonomy (CI: 1.06–80.77) compared to those born in Canada controlling for confounding variables. According to the MIST index, at least one of disrespectful behaviors was reported by 28.6% of immigrants and 35.8% Canada-born individuals (*p* = 0.172). Among immigrant participants, 2.6% reported being treated less favorably due to their ethnic, cultural, or linguistic background, compared to 1.5% of Canada-born participants (*p* = 0.09). Additionally, 9.1% of immigrants had to paid for obstetric care personally versus 4.4% of Canada-born (*p* = 0.173). Our study highlights immigration status as a key differentiating factor in obstetric care, with lower autonomy among temporary immigrants, while no significant differences were found between permanent immigrants and Canadian-born participants.

## Introduction

Disrespectful obstetric care is an increasingly recognized phenomenon worldwide. Studies show that disrespectful obstetric care and abuse are widespread, with mistreatment rates ranging from 17.3% in the United States [1] to almost universal levels in certain regions of Ethiopia [2]. Disrespectful obstetric care refers to medical practices and behaviors that do not respect a person’s rights during pregnancy, childbirth, and the postnatal period. They include verbal, physical, and emotional mistreatment, as well as unnecessary or harmful medical practices [3]. Disrespectful obstetric care can have negative consequences on a person’s health and well-being, increasing risks of trauma, complications, and maternal death, and negatively affecting the relationship between individuals and healthcare professionals [4]. Disrespectful obstetric care can deter individuals from seeking necessary medical care, leading some to avoid consulting doctors about pregnancy complications or to prefer unassisted home births [5]. Satisfaction, on the other hand, is defined as the overall contentment with care received during pregnancy, childbirth, and the postpartum period, considering various aspects such as the birth experience, healthcare professional interaction, and respect for dignity and privacy [6]. Access to obstetric care is another important dimension and includes the availability of services, geographical and economic accessibility [7].

Younger women, economically disadvantaged individuals, and women of color report higher rates of disrespectful obstetric care [8–10]. The available data in Quebec and Canada on obstetric care primarily focus on general dimensions such as maternal and infant mortality rates. However, these data often lack depth regarding women’s subjective experiences of obstetric care, particularly concerning mistreatment and the respectfulness of care received, especially among marginalized groups such as immigrants. Our study focuses on immigrant individuals, given the significant number of people who immigrate to Canada annually. According to the 2021 Census data, immigrants comprised 14.6% of Quebec’s population, with recent arrivals between 2016 and 2021 constituting 16.7% of this immigrant population [11]. Projections indicate that by 2036, immigrants could make up 24% to 30% of Canadian population, estimating that between 32% and 37% of Quebec’s population will fall into this category [12]. Slightly over half (5.8 million individuals) of these immigrants will be women, constituting 27.4% of Canada’s total female population [13].

Our study aims to comprehensively delineate the disparities in obstetric care experiences in Quebec between immigrant and Canada-born individuals, including disrespectful obstetric care, satisfaction regarding healthcare providers’ interpersonal skills and access to care. In this article, we adopt a gender-inclusive approach, recognizing that all individuals, regardless of gender identity, may need obstetrical services.

## Methods

### Study Design

This study is part of a greater research project conducted in Quebec, Canada, aiming to describe the experiences of obstetric and gynecological care in Quebec (PAROLES: “Pluralité et Amplification des voix pour le respect en obstétrique et gynécologie”). We report our findings following the Strengthening the Reporting of Observational Studies in Epidemiology (STROBE) guidelines [14].

This study was approved by the Committee for Research Ethics Involving Humans (CIEREH) of the University of Quebec at Montreal (UQAM) and by the Research Ethics Committee in Sciences and Health (CERSES) of the University of Montreal (UdeM). All data were anonymized.

### Study Population

The population of interest comprised individuals who received obstetric care in Quebec from 2016 to 2023. We included persons who received any type of obstetric care (prenatal care, labor and delivery, postnatal care) by healthcare professionals at least 3 months before completing the survey. Examples include obstetric ultrasounds, pregnancy follow-up visits, delivery, etc. Notably, abortion and first-trimester miscarriage were not included in obstetric care.

### Sampling

An online questionnaire was distributed using Qualtrics from July to December 2023. To ensure broad dissemination, it was published on social media, the project’s website, and the sites of CHU Sainte-Justine (Montreal, Canada) and partner associations. Additionally, flyers were distributed in associations and community centers to boost participation. This strategy aimed to reach a diverse audience and maximize response diversity. Participants were asked to complete the survey on the most recent episode of obstetric care they received. The development of our questionnaire was based on the RESPCCT study’s survey (Research Examining the Stories of Pregnancy and Childbearing in Canada Today) [8] and adapted to the Quebec context by a group of experts, including representatives from women’s rights advocacy groups, organizations advocating for respectful obstetric care, and an association of immigrant women from Quebec (Appendix 1). Participants were given the option to complete the questionnaires in French or English.

### Exposure

The main exposure of interest was immigration status at the time of obstetrical care, classified as follows: immigrants who were not born in Canada and hold temporary status (such as work permits, study permits, visitor visas, refugees, or asylum seekers) or permanent status (including permanent residents and Canadian citizens). The comparison group comprises participants who were born in Canada.

### Outcomes

The experience with the obstetrical care was measure using the following variables:

- The *Mothers’ Autonomy in Decision Making (MADM)* scale is a reliable instrument assessing autonomy in maternity care decision making. Developed and content validated with diverse groups of childbearing women in British Columbia, including vulnerable populations [8], it measures person’s ability to lead decisions, the time given to consider options, and whether choices are respected. Scores categorize autonomy as low (7–24), moderate (25–33), or high (34–42). In our sample, the MADM showed excellent reliability (Cronbach’s alpha = 0.95; 95% CI = 0.94–0.95), with item–total correlations from 0.76 to 0.84, indicating that each item contributed substantially to the construct. It has also demonstrated excellent reliability in previous studies [8, 15].
- Pregnant Persons Experience of Mistreatment by Providers Index (MIST): It is a set of patient-designed indicators providing a reliable and valid instrument to identify mistreatment [1]. Its seven components are: physical abuse, sexual abuse, verbal abuse, failure to meet professional care standards, poor interactions with providers, and poor conditions and constraints presented by the health system [16].In our sample, it demonstrated excellent internal consistency and reliability (Cronbach’s α = 0.93; 95% CI = 0.92–0.93), with item–total correlations ranging from 0.68 to 0.77, indicating that all items contributed adequately to the construct.
- Discrimination was examined more specifically through two variables. First, a binary variable assessed if participants felt they were treated worse than other patients due to their ethnic, cultural, or linguistic background. Second, another binary variable evaluated if they felt they were treated worse due to a cultural, traditional, or religious symbol they wore.
- Satisfaction with the interpersonal skills of healthcare professionals was evaluated using a categorical variable on a 6-point Likert scale (strongly satisfied to strongly dissatisfied). To simplify the analysis, it was further dichotomized into two categories: satisfied and dissatisfied.
- Access to healthcare was investigated based on two dimensions: financial and cultural, following the WHO conceptual framework [17]. The financial aspect was categorized into insured (provincial health card or private/group insurance) versus self-funded (personal or family funds). Cultural access was evaluated by asking whether participants received care from healthcare professionals sharing their ethnic background.

### Data Analysis

The MADM and MIST scales were compared between the two groups using bivariate chi-square and Fisher tests. For each scale, we restricted the analyze to the participants with a response rate of 85% and above, meaning they answered at least 6 out of the 7 items of the scale. Similarly, discrimination, satisfaction with healthcare providers’ interpersonal skills and access to care between immigrant and Canada-born persons were compared using chi-square.

A logistic regression was conducted to examine the association between having a low level of autonomy (MADM < 25) and immigration status while controlling for confounding variables. The explanatory variables included in the model were selected based on a literature review and encompassed the year care was received as it influences immigration status, the type of healthcare professional, maternal age, and satisfaction. Another logistic regression was conducted to examine the relationship between satisfaction regarding the interpersonal skills of healthcare providers and immigration status, while controlling for age, year and region of receiving care, type of healthcare professional, and autonomy levels. For the logistic regression models, both the Variance Inflation Factor (VIF) and McFadden’s pseudo R² was used to evaluate different aspects of model fit. The VIF assesses the extent of multicollinearity among the independent variables. A VIF of 1 indicates no collinearity between the variable and the other predictors in the model, allowing for confident interpretation of regression coefficients [18]. On the other hand, McFadden’s pseudo R² assesses the overall quality of model fit to the data with McFadden himself suggested that values between 0.2 and 0.4 denote an excellent fit [19].

The data were analyzed using R software, version 4.3.0.

## Results

### Sample Characteristics

Among 691 participants to the study, we excluded 5 participants who did not answer the question regarding their status as Canada-born or immigrant. Thus, we analyzed a sample of 686 participants, comprising 609 individuals born in Canada (88.8%) and 77 immigrants (11.2%). Compared to participants born in Canada, immigrants tended to be older, with a median age of 35 years compared to 32 years. Additionally, immigrants were more likely to have a higher level of education, with 49.4% holding a university degree (second or third cycle) compared to 25.8% of those born in Canada. However, 10.4% of immigrants reported an insufficient household income, compared to 5.6% of individuals born in Canada. Regarding other characteristics such as marital status, gender identity, and sexual orientation, no significant differences were observed between the two groups. Moreover, immigrants were more likely to have received obstetric care in high density regions (Montreal/Monteregie) (46.7%) than Canadian-born individuals (24.6%) and were more likely to receive care by midwives than by family physicians or other professionals (Table 1).

**Table 1:** Socio-demographic characteristics of the study sample (*n* = 686)

Characteristics	Immigrant participants (*N* = 77)	Participants Born in Canada (*N* = 609)	*P*-value
	*n* (%)	*n* (%)	
*Age (years)*			
Median (Q1, Q3)	35 (32, 38)	32 (29, 36)	< 0.001
*Level of Education*			
Primary, secondary, college, vocational, or trade school studies	12 (15.6)	220 (36.1)	< 0.001
Undergraduate studies	27 (35.0)	229 (37.6)	
Graduate studies	38 (49.4)	157 (25.8)	
Missing	0 (0.0)	3 (0.5)	
*Marital Status*			
Single	10 (13.0)	76 (12.5)	0.435
In a couple	66 (85.7)	519 (85.2)	
Other	0 (0.0)	13 (2.3)	
Missing	1 (1.3)	1 (0.2)	
*Gender Identity*			
Woman	74 (96.1)	586 (96.2)	1
Gender diversity	2 (2.6)	22 (3.6)	
Missing	1 (1.3)	1 (0.2)	
*Sexual Orientation*			
Heterosexual	67 (87.0)	526 (86.4)	1
Sexual diversity	10 (13.0)	76 (12.5)	
Missing	0 (0.0)	7 (1.1)	
*Minority Status*			
Racial/Ethnic Minority or Indigenous	23 (28.9)	68 (11.2)	< 0.001
Person with a Disability	1 (1.3)	24 (3.9)	0.345
Other	1 (1.3)	5 (0.8)	1
Not Identified as Minority	44 (57.1)	413 (67.8)	0.512
*Language Spoken at Home*			
French	64 (83.1)	497 (81.6)	< 0.001
English	6 (7.8)	107 (17.6)	
Other	7 (9.1)	2 (0.3)	
Missing	0 (0.0)	3 (0.5)	
*Household Income*			
More than sufficient	25 (32.5)	204 (33.5)	0.242
Sufficient	43 (55.8)	367 (60.2)	
Insufficient	8 (10.4)	34 (5.6)	
Missing	1 (1.3)	4 (0.7)	
*Principal Healthcare Professional*			
Midwife	26 (33.7)	173 (28.4)	0.193
Obstetrician-Gynecologist	29 (37.7)	229 (37.6)	
Family Physician	10 (13.0)	134 (22.0)	
Nurse/Specialized nurse practitioner (SNP)	9 (11.7)	40 (6.6)	
Other	1 (1.3)	4 (0.7)	
Missing	2 (2.6)	29 (4.7)	
*Location of the obstetric care*			
High density regions	36 (46.7)	150 (24.6)	< 0.001
Moderate density regions	17 (22.1)	141 (23.1)	
Low density regions	9 (11.7)	177 (29.1)	
Missing	15 (19.5)	141 (23.2)	
*Year of Receiving Care*			
2016–2019	16 (20.8)	189 (31.0)	0.194
2020–2021	22 (28.6)	171 (28.1)	
2022–2023	34 (44.1)	227 (37.3)	
Missing	5 (6.5)	22 (3.6)	

Immigrants had a uniform distribution in terms of years spent in Canada, with about a quarter having been in the country for less than 5 years, and a similar proportion for each of the 5–10 years, 10–15 years, and over 15 years categories. At the time of obstetrical care, 68.9% had permanent status and 14.3% had temporary status, with no refugees or asylum seekers (status missing for 16.8%). The majority were born in Europe (64.9%), followed by Africa (13.0%), America (9.1%) and Asia (6.5%) (country of birth was missing for 6.5%).

### Autonomy in Decision Making

The results of the analysis of the MADM scale between immigrant and Canada-born participants showed no significant difference and similar percentages were noted across all three levels of autonomy (Table 2).

**Table 2:** Mothers autonomy in decision making (MADM) scale according to immigrant and Canadian-born groups (*n* = 686)

	Immigrant participants (*N* = 77) *n* (%)	Participants born in Canada (*N* = 609) *n* (%)	*P*-value
Low Autonomy	14 (18.2)	124 (20.4)	0.903
Moderate Autonomy	23 (29.9)	177 (29.1)	
High Autonomy	38 (49.4)	292 (47.9)	

**Fig. 1:**
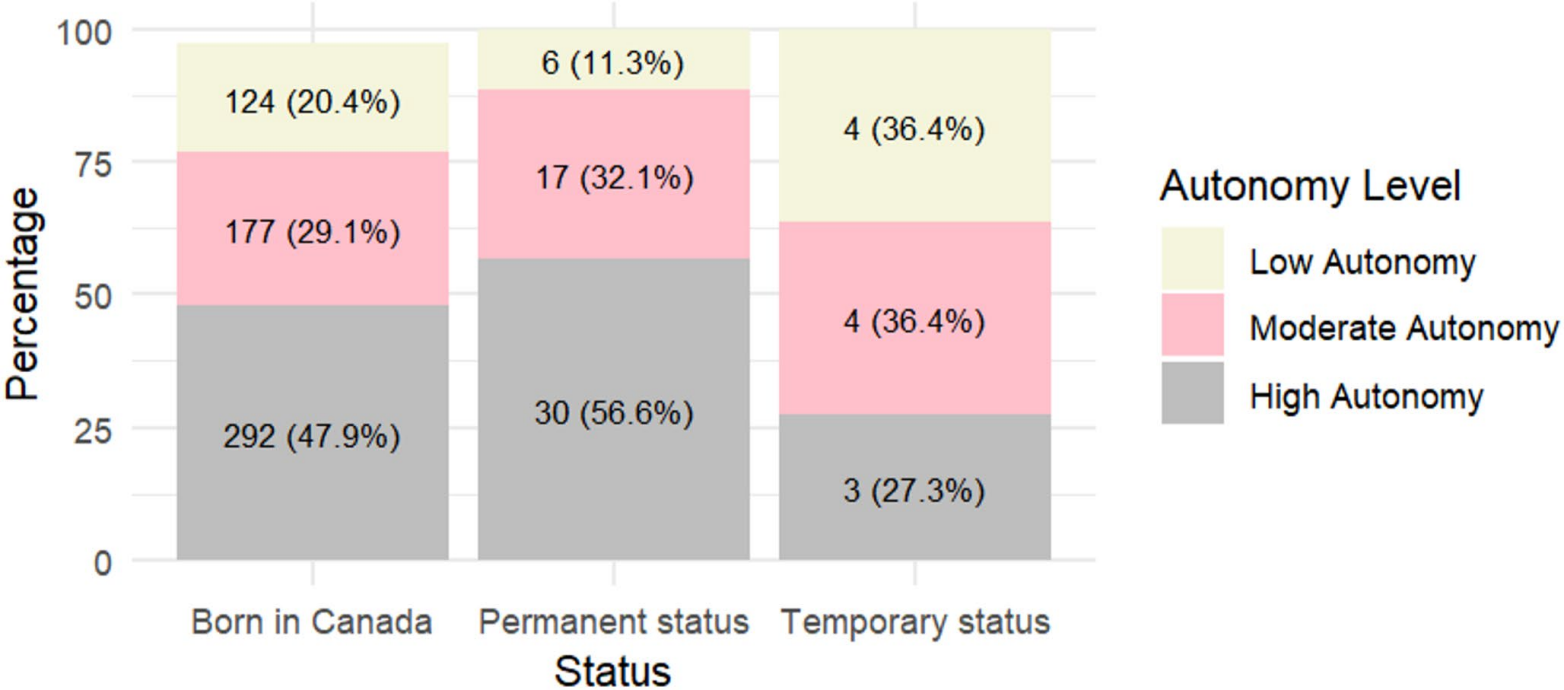
Levels of autonomy in decision making according to immigration status

Comparative analysis of immigrant subgroups reveals a non-significant disparity in autonomy levels (Fig. 1): among participants with permanent status, 56.6% exhibit a high level of autonomy, whereas 27.3% of those with temporary status fall into this category. Conversely, 36.4% of individuals with temporary status have a low level of autonomy, compared to 11.3% of those with permanent status.

After adjusting for age, year of receiving care, type of healthcare professional, and satisfaction, there was no significant difference in autonomy between immigrants with permanent status and participants born in Canada (OR = 0.68, 95% CI [0.25–1.84]). Conversely, individuals with temporary status were 9.25 times (95% CI [1.06–80.77]) more likely to have low autonomy compared to those born in Canada (Table 3). In the model, age, satisfaction with the interpersonal skills of healthcare professionals, receiving obstetrical care in 2022/2023, and being treated by a midwife were significantly associated with a reduced likelihood of experiencing low autonomy.

**Table 3:** Logistic regression of low autonomy level according to the immigration status (Moderate/High autonomy as reference)

Independent Variables	Odds Ratio	95% Confidence Interval	*P*-value
*Immigration status*			
Born in Canada	—	—	—
Immigrant with permanent status	0.68	0.25–1.84	0.45
Immigrant with temporary status	9.25	1.06–80.77	0.044
Age (years)	0.95	0.90–0.99	0.044
*Satisfaction with the interpersonal skills of healthcare professionals*			
Unsatisfied	—	—	—
Satisfied	0.11	0.06–0.20	< 0.001
*Year of Receiving Care*			
2016–2019	—	—	—
2020–2021	0.72	0.38, 1.37	0.32
2022–2023	0.41	0.21, 0.79	0.008
*Healthcare Professional*			
Midwife	—	—	—
Obstetrician-Gynecologist + Family Physician	9.40	3.97–22.28	< 0.001
Nurse/SNP + Other professionals	3.30	0.98–11.04	0.053

VIF values for all variables range from 1 to 1.1, indicating no issues with multicollinearity; McFadden’s pseudo R² of 0.46, suggesting a good fit

### Mistreatment

Among immigrant participants, 28.6% reported experiencing at least one of the forms of mistreatment, compared to 35.8% of Canada-born participants (p: 0.173). Additionally, the percentages of almost all forms of mistreatment were higher among Canada-born participants compared to immigrants (Table 4).

**Table 4:** Mistreatment according to immigrant status, MIST index (*n* = 686)

Have you experienced any of the following problems or behaviors during your obstetric care?	Immigrant participants (*N* = 77) % (*n*)	Participants born in Canada (*N* = 609) % (*n*)
Your private or personal information was shared without your consent	7.8 (6)	6.2 (38)
Your physical privacy was violated, for example being uncovered or having people in the delivery room without your consent	1.3 (1)	8.0 (49)
A healthcare provider shouted at or scolded you	11.6 (9)	15.8 (96)
Healthcare providers withheld treatment or forced you to accept treatment that you did not want	6.5 (5)	11.7 (71)
Healthcare providers threatened you in any other way	10.4 (8)	13.6 (83)
Healthcare providers ignored you, refused your request for help or failed to respond to requests for help in a reasonable amount of time	16.9 (13)	21.5 (131)
You experienced physical abuse, such as aggressive physical contact, inappropriate sexual conduct, a refusal to provide anesthesia for an episiotomy, etc.	7.8 (6)	13.5 (82)

### Discrimination

Among immigrant participants, 2.6% reported being treated less favorably due to their ethnic, cultural, or linguistic background, compared to 1.5% of Canada-born participants (p: 0.09). Additionally, in both groups, 1.3% stated being treated less favorably due to a cultural, traditional, or religious symbol they wear (p: 0.379).

### Accessibility

Following the WHO framework [17], access was examined through financial and cultural dimensions. The percentage of immigrants who paid for their care out of pocket (*n* = 7, 9.1%) is over twice that of Canadian-born participants (*n* = 27, 4.4%, p: 0.173). Moreover, 53% (*n* = 323) of Canadian-born individuals had a health care provider sharing their ethnicity, compared to 32.5% of immigrants (*n* = 25, *p* < 0.001).

### Satisfaction

**Fig. 2:**
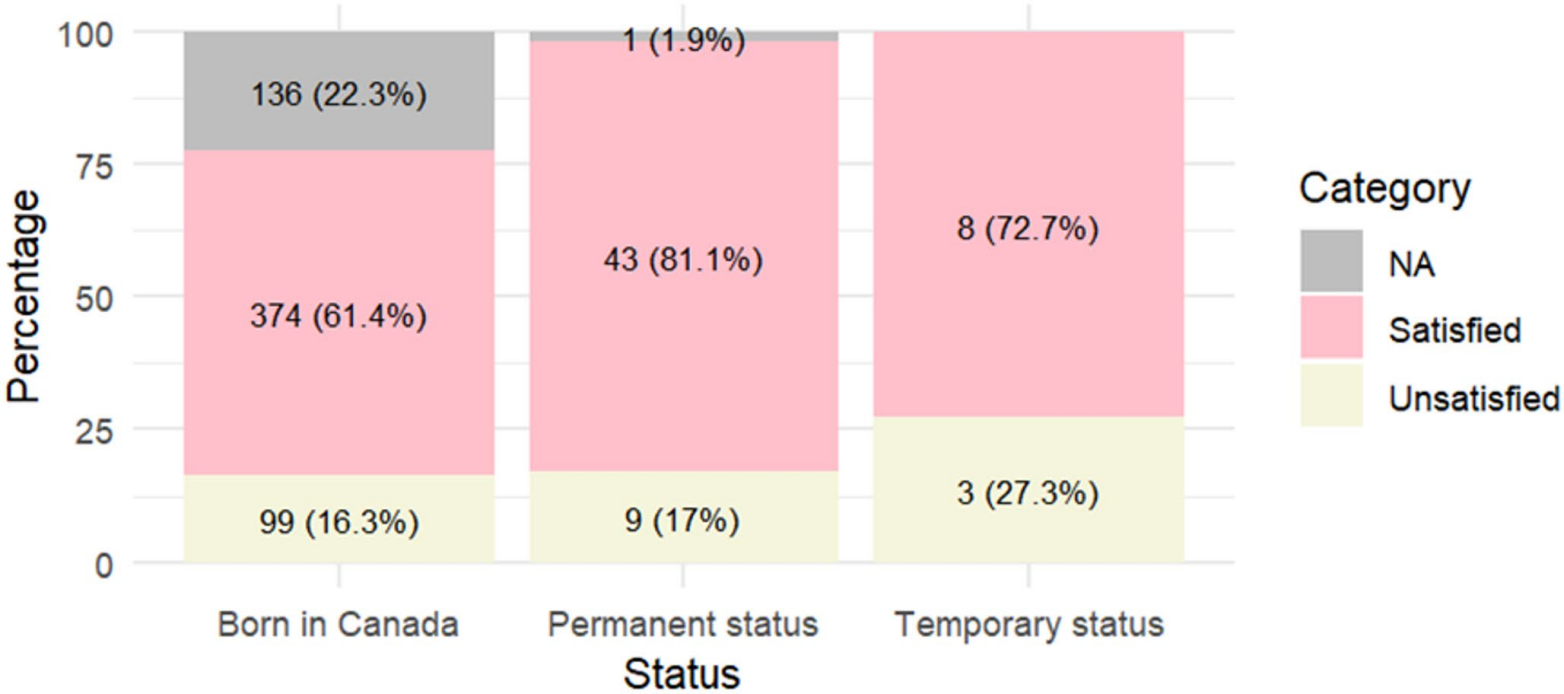
Satisfaction with healthcare providers’ interpersonal skills according to immigration status. *NA: not available

The percentages of satisfaction with healthcare provider’s interpersonal skills were similar between the Canada-born participants, participants with temporary status and participant with permanent status (Fig. 2).

After adjusting for age, year and region of receiving care, type of healthcare professional, and autonomy levels, there was no significant difference between the three groups (Table 5).

**Table 5:** Logistic regression of the satisfaction with healthcare professionals’ interpersonal skills according to the immigration status

Independent Variables	Odds Ratio	95% Confidence Interval	*P*-value
*Immigration status*			
Born in Canada	—	—	—
Immigrant with permanent status	0.87	0.35, 2.12	0.75
Immigrant with temporary status	0.80	0.12, 5.30	0.82
Age	1.04	0.99, 1.09	0.16
*Year of Receiving Care*			
2016–2019	—	—	—
2020–2021	1.40	0.75, 2.62	0.30
2022–2023	2.44	1.27, 4.69	0.007
*Healthcare Professional*			
Midwife	—	—	—
Obstetrician-Gynecologist or Family Physician	1.81	0.83, 3.97	0.14
Nurse/SNP or Other professionals	0.58	0.20, 1.67	0.31
*Autonomy level*			
High Autonomy	—	—	—
Moderate Autonomy	0.21	0.09, 0.46	< 0.001
Low Autonomy	0.04	0.02, 0.09	< 0.001
*Region of Care*			
Moderate density regions	—	—	—
High density regions	1.98	1.03, 3.80	0.040
Low density regions	2.64	1.36, 5.12	0.004

VIF values for all variables range from 1 to 1.8, indicating no issues with multicollinearity; McFadden’s pseudo R² =0.32, suggesting a good overall quality of fit for the model to the data

## Discussion

Our findings indicate some differences between immigrants and Canadian-born individuals regarding obstetric care experiences, especially autonomy and access to care. Immigrants with temporary status exhibited significantly lower autonomy compared to Canadian-born individuals. Also, they had lower access to health insurance coverage and to healthcare professionals sharing their ethnicity.

Other studies also found lower autonomy in immigrant population, without differentiating by immigration status. Immigrants may cede their autonomy in decisions regarding childbirth, viewing the medical system as the authority on power, knowledge, and decision-making. In a study conducted in Chile, immigrant women often assume passive roles in healthcare settings, relying on professionals’ expertise and hesitating to express their own preferences or concerns [20]. This pressure to prioritize pleasing caregivers over personal needs fosters a dynamic of “grateful submission,” where autonomy is sacrificed for perceived supportive care. For example, some women may feel coerced into accepting anesthesia, challenging their right to manage pain naturally and straining relationships with providers who disapprove of their refusal [20]. Additionally, the factors influencing shared decision-making among healthcare providers can be summarized into three main elements: providers’ motivation, belief in the positive outcomes of shared decision-making, and its perceived beneficial impact on the clinical process [21]. Therefore, if providers are not motivated or do not believe in the benefits of shared decision-making, this can further reduce the autonomy of immigrants. On the other hand, understanding that shared decision-making improves clinical outcomes can enhance care quality and support immigrant’s autonomy. Addressing these factors among healthcare providers is key to improving the obstetric care experiences and autonomy of immigrants.

Our results also show that higher levels of autonomy were significantly correlated with greater satisfaction, highlighting the crucial role of patient autonomy in healthcare experiences. In fact, for individuals with moderate or low autonomy, their chances of being satisfied with healthcare professionals’ interpersonal skills were reduced by 79% and 96%, respectively (*p* < 0.001), compared to those with high autonomy. Conversely, being satisfied with healthcare professionals’ interpersonal skills reduced the likelihood of having low autonomy by 89% (*p* < 0.001).

Moreover, encouraging patients to play a significant role in medical decision-making offers major benefits: increased satisfaction, a reduction in excessive interventions, and improved sustainability of healthcare systems. This fosters a positive dynamic where care becomes more patient-centered, and resources are utilized more effectively [22].

Regarding mistreatment, 28.6% of immigrant individuals in Quebec reported experiencing at least one disrespectful behavior, compared to 35.8% of Canadian-born individuals, although this difference was not statistically significant, the observed trends provide valuable insights into patients’ experiences. They may point to underlying factors related to immigration status that warrant further investigation. Similarly, a study conducted in Iceland found no significant differences between migrant and native-born women; overall, about 24% (*n* = 328) of participants reported experiencing at least one form of mistreatment, and 8.9% (*n* = 121) reported two or more forms [23]. This percentage is higher in the United States, where one in six women (17.3%) has experienced one or more types of mistreatments [1]. Examining physical violence during obstetric care more specifically, 7.8% of immigrants reported experiencing physical violence during such care, compared to 13.5% of Canadian-born individuals. Similarly, in Chile, the majority of immigrant women reported receiving the same treatment as native-born women and did not experience the mistreatment they had feared. Studies on mistreatment of immigrants during obstetric care are very limited in Canada and primarily focus on mistreatment experienced outside healthcare facilities. It is noted that immigrants often report more favorable healthcare experiences compared to non-immigrants because they generally have lower expectations based on their experiences in countries with fewer resources or different care standards, though this can vary significantly depending on their country or region of origin [24]. Consequently, when the healthcare in their new country exceeds these expectations, they perceive it more positively [24]. Studies support this phenomenon, showing a significant contrast between the healthcare in their home and host countries, leading to better perceptions [24, 25]. According to a study conducted with recently immigrated South Asian women in Montreal, most participants were surprised by Montreal hospitals promoting vaginal births, contrasting this with the Bangladeshi protocol that prioritizes doctors’ agendas over birthing women’s needs, making cesarean sections feel obligatory. In Montreal, however, vaginal birth is seen as a way for women to reclaim their birthing experience, as they become the central figures in the process [26].

Regarding economic access to obstetrical care, although there were no significative differences, 9.1% of immigrants, compared to 4.4% of those born in Canada, used out-of-pocket payments from personal or family funds. All immigrant categories face barriers accessing care in Canada [27]. The lack of insurance among immigrants creates an internalized sense of not being worthy or deserving of care. As a result, they often delay or avoid seeking healthcare, leading to negative outcomes such as worsening chronic conditions, inadequate or delayed prenatal care, and improper medication use [28]. Some studies show that immigrant women suffer from inequities in sexual and reproductive health care access, which is further compounded by perceptions of the Canadian health system as inconvenient and inadequate due to institutional barriers such as difficulties finding a family doctor, long wait times, quick hospital discharges, limited health insurance coverage, and high costs [29]. A cross-sectional study conducted in 2014–2015 among immigrant women (*N* = 2636) giving birth in four Montreal hospitals found that nearly one in four participants (22.9%) reported barriers to accessing services during pregnancy, while only 11% or fewer were asked about their care preferences [30]. Similarly, in Chile, compared to local residents, immigrants were 7.5 times more likely not to have insurance. In addition to a greater lack of appointments, coverage, and unmet needs, immigrants were perceived as having fewer needs than natives [31].

### Strengths

Our study is among the first investigations into the experience of obstetric care of immigrant people in Quebec and highlighting a critical yet often overlooked aspect of healthcare. By using social media for recruitment, we achieved broad participation, assembling a diverse cohort of individuals who had experienced obstetric care in the province. To address the challenge of excluding persons who lack internet access or literacy skills, community organizations played a crucial role by assisting participants in completing questionnaires at their locations.

### Limitations

Despite the support of a communication agency to enhance outreach efforts, aiming for representation across Quebec’s diverse population, particularly focusing on marginalized groups, the external validity of the study is limited. Indeed, the noticeable absence of refugees and asylum seekers in our sample underscores a significant gap in our comprehension. Moreover, persons who had midwife as principal health care provider were overrepresented in our sample: among the participants, 31.1% had obstetric care provided by midwives, while the provincial rate is 4.5% [32]. In addition, although 68% of participants were French speakers, and responses were collected in the language of their choice, we did not examine potential differences based on language, which may further limit generalizability. Furthermore, our sample shows a significant overrepresentation of immigrants born in Europe compared to the general immigrant population in Quebec, while those born in Africa, Asia, and America are notably underrepresented [11]. The differences in representation indicate a selection bias. The overrepresentation and underrepresentation of certain groups mean that the experiences reported in our study may not fully reflect the true diversity of obstetric care experiences among all immigrants in Quebec. Consequently, this selection bias from non-representative sampling primarily limits our study’s external validity. These discrepancies limit the ability to generalize the results to the entire population in Quebec.

## Conclusion and Future Directions

Our findings underscore the importance of addressing disparities in decision-making autonomy and financial access to care among immigrant populations in Quebec, with a particular focus on individuals with temporary status. Interventions aimed at reducing financial barriers to healthcare access among immigrant populations should be developed and evaluated to promote equitable access to healthcare services. By promoting culturally competent and patient-centered care practices, healthcare systems can strive towards equitable healthcare experiences for all individuals, irrespective of their immigration status. Future research should aim to include a more diverse sample that encompasses refugees, asylum seekers, and individuals from a wider range of socioeconomic backgrounds to better understand the experiences of these vulnerable populations.

Overall, our findings contribute to a better understanding of the healthcare experiences of immigrants in Quebec and highlight the need for targeted interventions to address disparities in healthcare access and promote patient-centered care for all populations.

### New Contribution To the Literature

This study highlights differences in the experience of obstetric care between immigrant and Canada-born individuals in Quebec, revealing that while immigrants report less mistreatment, they face greater challenges in accessing health insurance, with those holding temporary status experiencing lower autonomy.

## Data Availability

No datasets were generated or analysed during the current study.

## Appendix

Our collaborators come from various institutional and clinical practice backgrounds:

Here is the list translated into English:

- Regroupement Naissance-Respectée (Respected Birth Group).
- CHU Sainte-Justine.
- L’R des centres de femmes du Québec (L’R Women’s Centers of Quebec).
- Réseau des centres de ressources périnatales du Québec (Quebec Perinatal Resource Centers Network).
- Fédération québécoise pour le planning des naissances (FQPN) (Quebec Federation for Birth Control).
- Réseau d’action des femmes handicapées du Canada (RAFH) (Canadian Network of Women with Disabilities).
- Réseau d’action pour l’égalité des femmes immigrées et racisées du Québec (RAFIQ) (Network for Action on Equality for Immigrant and Racialized Women of Quebec).
- Le Mouvement pour l’autonomie dans la maternité et l’accouchement naturel (MAM) (Movement for Autonomy in Maternity and Natural Childbirth).
- Regroupement Les Sages-Femmes du Québec (RSFQ) (Quebec Midwives Association).
- Ordre des Sages-femmes du Québec (OSFQ) (Order of Midwives of Quebec).
- Association québécoise des infirmières et infirmiers (AQII) (Quebec Association of Nurses).
- Groupe de spécialistes en Médecine fœto-maternelle du Québec (Group of Specialists in Fetal-Maternal Medicine of Quebec).
- Association des omnipraticiens en périnatalité du Québec (AOPQ) (Association of Perinatal General Practitioners of Quebec).
- Association des obstétriciens et gynécologues du Québec (AOGQ) (Association of Obstetricians and Gynecologists of Quebec).
- Ordre des infirmières et infirmiers du Québec (OIIQ) (Order of Nurses of Quebec).
- Collège des médecins du Québec (College of Physicians of Quebec).
